# Effects of increasing intake of soybean oil on synthesis of testosterone in Leydig cells

**DOI:** 10.1186/s12986-021-00580-1

**Published:** 2021-05-26

**Authors:** Yu Su, Zhenhua Tian, Xiangyu Qi, Dandan Luo, Luna Liu, Shuang Liu, Dongmei Zheng, Fang Wei, Zhao He, Qingbo Guan

**Affiliations:** 1 Department of Endocrinology and Metabolism, Shandong Provincial Hospital, Cheeloo College of Medicine, Shandong University, Jinan, 250021 Shandong China; 2 Shandong Provincial Key Laboratory of Endocrinology and Lipid Metabolism, Institute of Endocrinology and Metabolism, Shandong Academy of Clinical Medicine, Jinan, 250021 Shandong China; 3 Department of Endocrinology and Metabolism, Shandong Provincial Hospital Affiliated to Shandong First Medical University, Jinan, 250021 Shandong China; 4 Department of Clinical Expert, Shandong Provincial Hospital Affiliated to Shandong First Medical University, Jinan, 250021 Shandong China

**Keywords:** Soybean oil, Linoleic acid (LA), α-linolenic acid (ALA), Testosterone, Luteinizing hormone (LH)

## Abstract

**Background:**

Soybean oil is a very common edible oil in daily life. With the changes in the dietary composition, the intake of soybean oil increased. However, the effects of dietary intake of soybean oil on testosterone production are still unclear.

**Methods:**

In order to study the effects of increasing intake of soybean oil on the synthesis of testosterone in Leydig cells, we fed male C57BL/6 mice on the diet which added 20% soybean salad oil (SOY group). We detected the hormone levels by enzyme-linked immunosorbent assay (ELISA) kits and serum fatty acid composition by gas chromatography, and analyzed the expression of steroidogenic enzymes by Real-Time PCR or immunoblotting analysis.

**Results:**

After the 16-week feeding period, serum linoleic acid (LA) and α-linolenic acid (ALA) significantly increased and serum palmitic acid (PA) significantly decreased in SOY group mice. Compared to the normal diet (ND group), increasing intake of soybean oil raised the luteinizing hormone (LH) levels and up-regulated luteinizing hormone/chorionic gonadotropin receptor (LHCGR), steroidogenic acute regulatory protein (StAR) and cytochrome P450 family 11 subfamily A member I (CYP11A1). Testosterone levels in SOY group were higher than that in the ND group, and significantly difference showed.

**Conclusions:**

Increasing intake of soybean oil could raise the serum LA and ALA levels and decrease serum PA levels. This could activate the LH/LHCGR pathway and improve the function of steroid synthesis in Leydig cells, and finally lead to the elevated testosterone levels.

**Supplementary Information:**

The online version contains supplementary material available at 10.1186/s12986-021-00580-1.

## Introduction

Testosterone (T), as an important steroid hormone, plays a key role in the physiological processes of male growth and development, maintenance of fertility, and material metabolism [1–3]. A cross-sectional survey reported that testosterone levels declined 25% among adolescent and young adult men in the USA in 2015–2016 compared to sixteen years ago [4]. The lack of testosterone leads to male sexual dysfunction, accelerates the aging of the body’s organs, induces the occurrence of systemic diseases, and thus, seriously affects the quality of life [5, 6]. Therefore, the decline in testosterone levels is a major concern of the public health. It has significant meanings to explore the underlying mechanism and potential treatment of testosterone level decline.

Polyunsaturated fatty acids (PUFAs) are nutrients required for a variety of physiologic functions that exert important health effects. A nationwide cross-sectional survey of Koreans shows that soybean oil was the major source of PUFAs [7]. Soybean oil is a very common edible oil in daily life. Some studies have revealed that soybean oil improves myocardial function and it has anti-oxidation effect [8, 9]. The main component of soybean oil is linoleic acid (C18:2 ω-6, LA), it is a kind of ω-6 PUFAs [10]. In addition, compared with other edible oils, soybean oil is rich in α-linolenic acid (C18:3 ω-3, ALA) [11]. LA and ALA are essential because they cannot be synthesized by humans and must be obtained through diet, primarily from plant oils [12]. In the United States, there has been a rapidly 1000-fold increase in the consumption of soybean oil during the twentieth century [13]. But the effect of increasing intake of soybean oil on testosterone synthesis and its underlying mechanisms remains unknown.

Male testosterone is mainly produced by testicular Leydig cells, and is regulated by the hypothalamic-pituitary axis. After luteinizing hormone (LH) acting on the luteinizing hormone/chorionic gonadotropin receptor (LHCGR) on Leydig cells, cholesterol is transported into the mitochondria via the Steroidogenic acute regulatory protein (StAR) [14], which is considered as the rate-limiting step in testosterone synthesis [15], and finally converted to testosterone by steroidogenic enzymes such as cytochrome P450 family 11 subfamily A member 1 (CYP11A1), 3β-hydroxysteroid dehydrogenase (3β-HSD) and cytochrome P450 family 17 subfamily A member 1 (CYP17A1) [16, 17]. Increasing intake soybean oil may affect testosterone production through one or more points in the process mentioned above.

In our study, we established an animal model by adding soybean oil to the diet to compare the effects of increasing intake of soybean oil on testosterone synthesis. This study suggests that increasing intake of soybean oil could activate the LH/LHCGR pathway and promote testosterone production.

## Methods

### Animals and treatment

All animal experiments were approved by the Animal Ethics Committee of Shandong Provincial Hospital and performed according to the Shandong Provincial Hospital Animal Care and Use Committee. Fourteen male C57BL/6 mice at 6–7 weeks of age were constructed from Beijing Vital River Laboratory Animal Technology Co., Ltd. (Beijing, China). They were housed in a constant temperature-controlled room for a 12-h light/dark cycle. After acclimated for one week, they were divided into two groups and assigned to one of the following three diets for 16 weeks: (1) normal diet (ND group), (2) soybean oil diet (SOY group). All the diets were provided by Beijing Keaoxieli Co., Ltd (Beijing, China). The normal diet provided calories of 3.40 kcal/g (protein 23.07%, fat 11.85%, carbohydrate 65.08%). Additional file 1: Table S1 shows the ingredients of normal diet. The soybean oil diet was prepared by supplementing normal diet with about 20% soybean salad oil (by weight), it was providing calories of 5.36 kcal/g (protein 14.62%, fat 44.13%, carbohydrate 41.25%). The fatty acid compositions of soybean oil and the diets are shown in Additional file 2: Table S2 and Additional file 3: Table S3. To measure food intake, we separated animals in single cages for 7 days. A weighed amount of food was given and the weight consumed (evaluated as the difference between the original amount and the food left in the cage) was measured per-day. The mice were killed for the subsequent experiments after 16 weeks.

### Measurement of lipid profile and sex hormone

At the 16th week, after being fasted for 8 h, the mice were anaesthetized with pentobarbital sodium and the blood samples were collected from eyes. The blood was centrifuged to separate the mice serum. Triglyceride (TG), total cholesterol (TC), low-density lipoprotein cholesterol (LDL-C), high-density lipoprotein cholesterol (HDL-C) and glucose (Glu) was measured with biochemical analyzer (Mindray Bio Medical Electronics Co., Ltd.). Serum testosterone (CUSABIO Biotech Co., Ltd., Wuhan, China) and serum luteinizing hormone (Cloud-Clone Corp, Houston, USA) levels were measured by an enzyme-linked immunosorbent assay (ELISA) kit according to the manufacturer's protocols.

### Gas chromatographic analysis of fatty acids

Added 1% sulfuric acid methanol solution to the serum and esterified the samples at 80 °C. Then the samples were extracted with n-hexane and washed with ddH2O. Added anhydrous sodium sulfate powder to the supernatant to remove excess water. The samples were centrifuged to separate the supernatant. Methyl salicylate as an internal standard was added to the supernatant. Samples were measured by Agilent 6890N/5975B gas-mass spectrometer (Agilent, USA).

### RNA isolation and Real-time PCR

RNAiso Reagent (Takara, Tokyo, Japan) was used to extract total RNA from testicular tissues. RNA samples were reverse-transcribed by reverse transcription kit (PrimeScript® RT Reagent Kit (Perfect Real Time), TaKaRa, Japan) according to the manufacturer's instructions. The cDNA obtained by reverse transcription was used as a template for RT‐PCR conducted with the LightCycler480 (Roche). Use the 2^−ΔΔCt^ method to calculate the relative expression of mRNA. The sequences of the PCR primers (synthesized by Qingdaoqingke Biotech Co., Ltd., China) of LHCGR (5′- GATGCACAGTGGCACCTTC and 5′- TCAGCGTGGCAACCAGTAG), STAR (5′-GGAGCAGAGTGGTGTCATCAGA and 5′-AGGTGGTTGGCGAACTCTATCT), SF-1 (5′-CCCAAGAGTTAGTGCTCCAGT and 5′-CTGGGCGTCCTTTACGAGG), CYP11A1 (5′-TGCTTGAGAGGCTGGAAGTTGA and 5′-CGGATTGCGGAGCTGGAGAT), CYP17A1 (5′-GTACCCAGGCGAAGAGAATAGA and 5′-GCCCAAGTCAAAGACACCTAAT), 3β-HSD (5′-AGCTCTGGACAAAGTATTCCGA and 5′-GCCTCCAATAGGTTCTGGGT)and β-actin (5′-GGCTGTATTCCCCTCCATCG and 5′-CCAGTTGGTAACAATGCCATGT).

### Immunoblotting analysis

Total protein was extracted from testicular tissues with protein lysis buffer (RIPA: PMSF = 99:1), and determined the concentration by the BCA assay. After denaturation, protein samples were subjected to SDS-PAGE, electrophoretically transferred to membranes. The membrane was blocked in 5% skimmed milk in TBST buffer for 1 h, and then incubated with primary antibody overnight at 4 °C. StAR (1:1000) and CYP11A1 (1:1000) is from Cell Signaling Technology, 3β-HSD (1:500) is from Santa Cruz; LHCGR (1:500), CYP17A1 (1:1000) and GAPDH (1:5000) are from Proteintech. The membrane was incubated with the appropriate secondary antibodies for 1 h. Finally, we visualized and quantified the bands.

### Immunohistochemistry

The testes were fixed for 24 h and then embedded in paraffin. For the immunohistochemistry assay, all steps followed standard techniques. The paraffin sections were deparaffinized, hydrated, and antigen repaired. Then incubated them overnight at 4 °C with the primary rabbit anti-LHCGR primary antibody (Proteintech,1:200), and use BSA as a negative control. The next day, they were incubated with the secondary antibody at 37 °C, and then developed with DAB kit (ZSGB-BIO, Beijing, China) and counterstained with hematoxylin.

### Statistical analysis

All data were analyzed by GraphPad Prism (v.8, GraphPad Software Inc, CA). The intergroup comparison was determined by t-test. All data are expressed as mean ± SD, and it is statistically significant when *P* < 0.05.

## Results

### Effect of increasing intake of soybean oil on serum lipid profiles and glucose levels in the mice

**Fig. 1:**
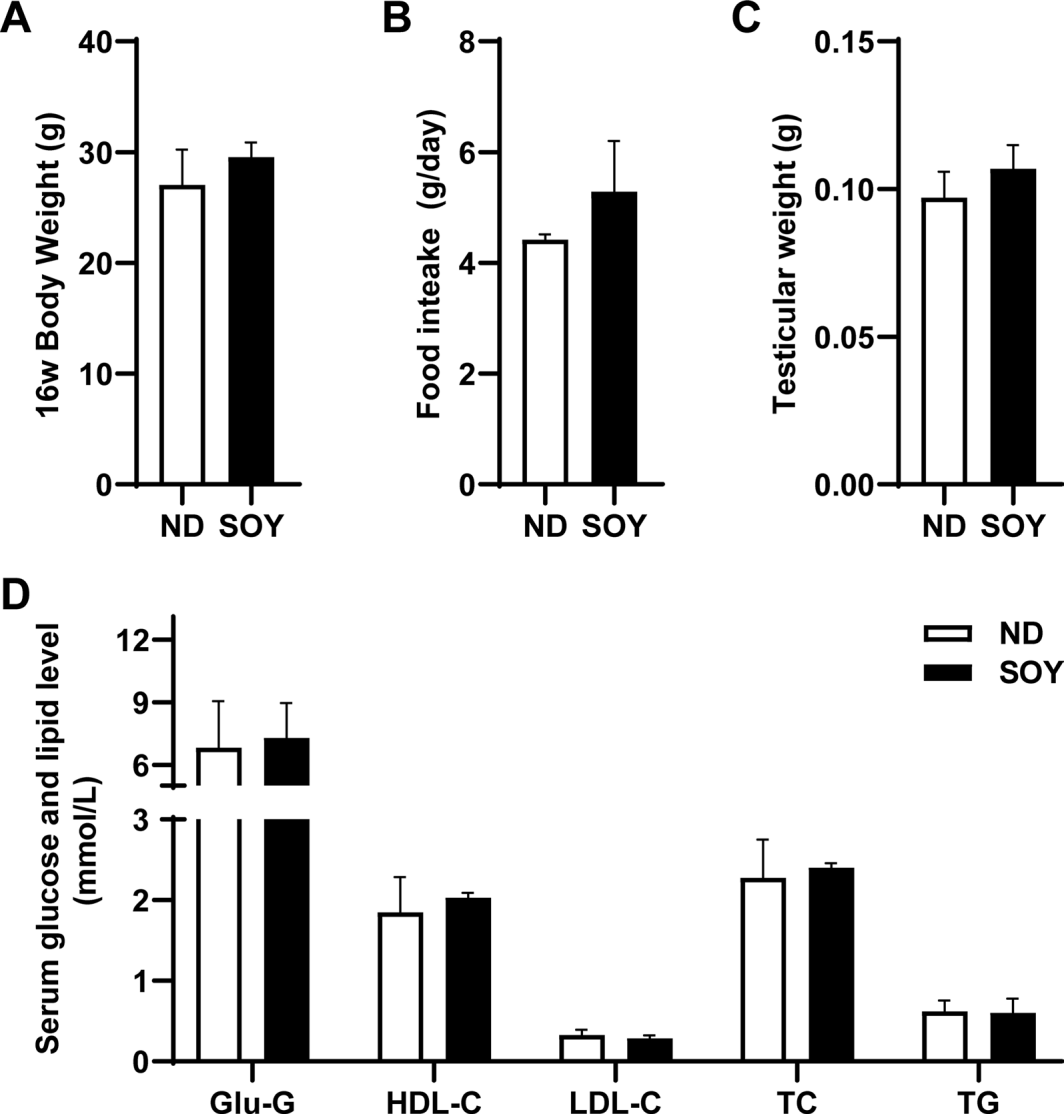
Effect of increasing intake of soybean oil on serum lipid profiles and glucose levels in the mice. **A** The body weight at the 16th week of mice fed with ND or SOY group (n = 7 for each group). **B** The amount of food intake of the ND or SOY group (n = 3 for each group). **C** The weight of testis in the ND or SOY group (n = 7 for each group). **D** Comparison of serum lipid levels and Glu levels in the ND or SOY group (n = 6 for each group). Data are expressed as mean ± SD

We measured the weight of the mice after the 16th weeks’ feeding. Comparing with the ND group, the body weight of the mice in the SOY group increased slightly, but it did not have statistical difference (Fig. 1A). There was no significant difference in food intake between the two groups (Fig. 1B). After the 16-week feeding period, no significant difference was seen in testicular weight among the two groups (Fig. 1C). To observe the effects of adding soybean oil on circulating blood lipids and glucose (Glu), we tested the serum levels of LDL-C, HDL-C, TC, TG and Glu. As shown in the Fig. 1D, increasing intake of soybean oil did not affect the blood lipid profiles and Glu levels; there was no significant difference among the two groups.

### Increasing intake of soybean oil influenced the serum hormone levels in the mice

**Fig. 2:**
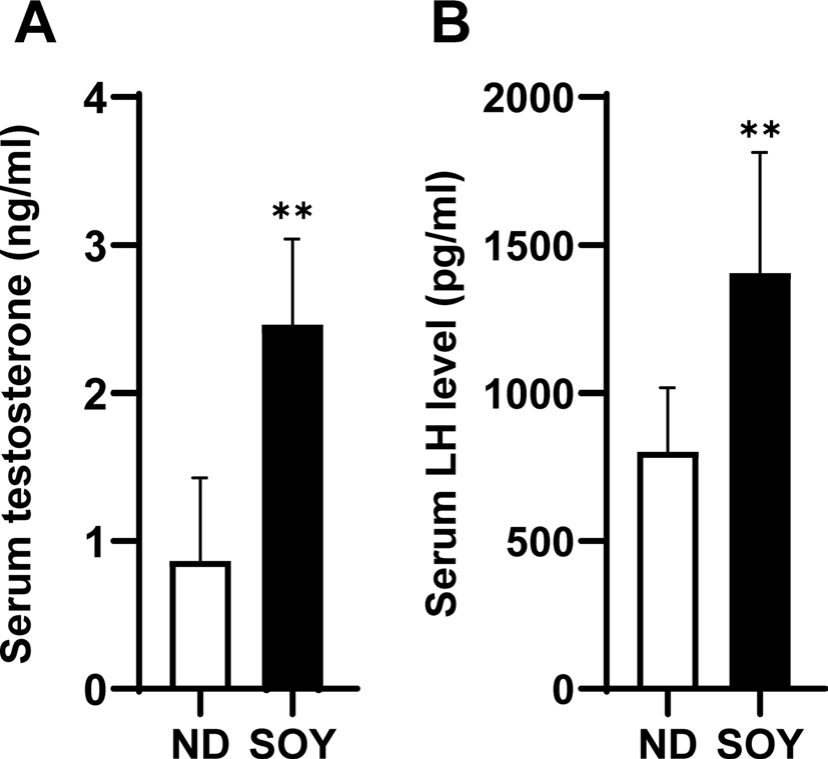
Increasing intake of soybean oil influenced the serum hormone levels in the mice. **A** The serum testosterone levels of mice in the ND or SOY group (n = 5 for each group). **B** The serum LH levels of mice in the ND or SOY group (n = 7 for each group). Data are expressed as mean ± SD (**P < 0.01)

In order to explore the effects of increasing intake of soybean oil on serum sex hormones, serum testosterone and LH levels were detected. The testosterone levels of the mice in SOY group were observed dramatically increasing compared with those of the ND group (Fig. 2A; *P* < 0.01). LH acts on Leydig cells and promotes the production of testosterone. The serum LH levels of mice in the SOY groups significantly increased compared with the ND group. (Fig. 2B). Increasing intake of soybean oil could increase the LH levels, and the high LH levels may further promote the production of testosterone.

### Increasing intake of soybean oil influenced the serum fatty acid composition in the mice

Increasing intake of soybean oil affected LH levels and testosterone production. In order to observe the effect of soybean oil on the serum fatty acid composition, we analyzed the serum by gas chromatography. Soybean oil is the source of LA and ALA. As shown in Table 1, compared with ND group, the serum LA and ALA levels in the mice of the SOY group were significantly increased. And the serum palmitic acid (C16:0, PA) and total saturated fatty acid were significantly decreased. Comparing with the ND group, with the percent of LA and ALA increased, the percent of C14:0, C14:1, C22:1, C24:1, C18:3, C20:4, C20:3, C20:5 and C22:6 declined in the SOY group. It is suggested that increasing intake of soybean oil may influence serum hormone levels by the changes of serum fatty acid composition.

**Table 1:** Analysis of the content of fatty acid (FA) in the serum

FA (%)	Group	
	ND	SOY
*Saturated FA*		
C14:0 (Myristic acid)	0.71 ± 0.07	0.62 ± 0.02*
C16:0 (PA)	56.27 ± 1.08	53.42 ± 0.98***
C18:0 (Stearic acid)	26.10 ± 0.66	26.51 ± 0.65
C20:0 (Arachidic acid)	0.46 ± 0.03	0.45 ± 0.02
C22:0	0.15 ± 0.01	0.14 ± 0.01
Total saturated FA	83.68 ± 1.55	81.13 ± 1.60*
*Monounsaturated FA*		
C14:1	0.31 ± 0.04	0.26 ± 0.03*
C16:1 (Palmitoleic Acid)	0.65 ± 0.13	0.51 ± 0.08
C18:1 (Oleic acid)	1.79 ± 0.22	1.91 ± 0.38
C20:1	0.29 ± 0.03	0.26 ± 0.02
C22:1 (Erucic acid)	0.47 ± 0.02	0.43 ± 0.04*
C24:1	0.28 ± 0.02	0.23 ± 0.02**
Total monounsaturated FA	3.79 ± 0.22	3.60 ± 0.44
*ω-6 Polyunsaturated FA*		
C18:2 ω-6 (LA)	7.35 ± 0.98	11.09 ± 0.99***
C18:3 ω-6 (γ-Linolenic acid)	0.22 ± 0.02	0.15 ± 0.02***
C20:3 ω-6	0.29 ± 0.07	0.25 ± 0.03
C20:4 ω-6 (AA)	2.94 ± 0.48	2.33 ± 0.43*
Total ω-6 polyunsaturated FA	10.81 ± 1.40	13.82 ± 1.20**
*ω-3 Polyunsaturated FA*		
C18:3 ω-3 (ALA)	0.25 ± 0.03	0.42 ± 0.08***
C20:3 ω-3	0.32 ± 0.01	0.29 ± 0.03*
C20:5 ω-3 (EPA)	0.28 ± 0.03	0.17 ± 0.02***
C22:6 ω-3 (DHA)	0.86 ± 0.14	0.57 ± 0.07***
Total ω-3 polyunsaturated FA	1.71 ± 0.17	1.45 ± 0.11*

n = 6 for each group. Data were presented as mean ± SD (*P < 0.05; **P < 0.01; ***P < 0.001)

PA: Palmitic acid; LA: Linoleic acid; AA: Arachidonic acid; ALA: α-linolenic acid; EPA: Eicosapentaenoic acid; DHA: Docosahexaenoic acid

### Increasing intake of soybean oil enhanced steroidogenic enzyme expression in the testis

**Fig. 3:**
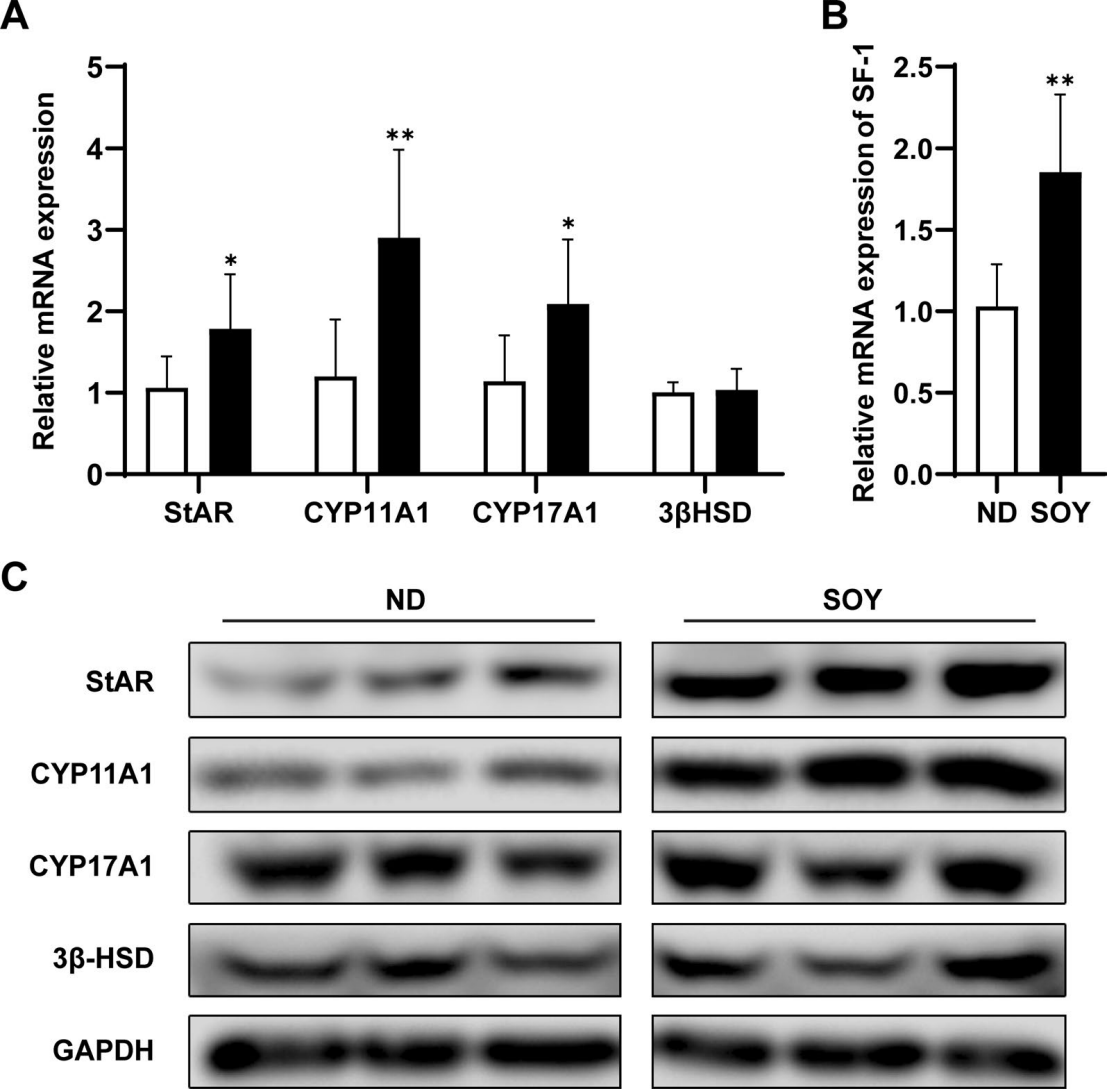
Increasing intake of soybean oil enhanced steroidogenic enzyme expression in the testis. **A** RT‐PCR analysis of the expression of StAR, CYP11A1, CYP17A1 and 3β‐HSD mRNA in testes from mice in the ND or SOY group (n = 6 for each group). **B** RT‐PCR analysis of the expression of SF-1 mRNA in testes from mice in the ND or SOY group (n = 6 for each group). **C** Immunoblot and quantitative analysis of StAR, CYP11A1, CYP17A1 and 3β‐HSD protein in testes of mice in the ND or SOY group (n = 3 for each group). Data are expressed as mean ± SD (*P < 0.05, **P < 0.01)

Testosterone synthesis is a complex process in which the key enzymes StAR, CYP11A1, CYP17A1, and 3β-HSD are involved. In order to explore the effect of increasing intake of soybean oil on the enzymes related to the testosterone synthesis pathway of Leydig cells, we used RT-PCR and immunoblotting analysis to detect the expression of these proteins. Compared with the ND group, the mRNA levels of StAR, CYP11A1, and CYP17A1 in the SOY group all significantly increased (Fig. 3A). There was no difference between the two groups of 3β-HSD mRNA levels. We also tested the mRNA levels of steroidogenic factor 1 (SF-1), which could promote the transcription of testosterone synthesis related genes such as StAR, CYP11A1 and 3β-HSD [18, 19]. We found that the mRNA levels of SF-1 in the SOY group significantly increased compared to ND group. As shown in Fig. 3C, consistent with the mRNA levels, the protein expression of StAR and CYP11A1 increased in the SOY group in comparison to ND group. In Fig. 3, no significant difference about CYP17A1 at the protein levels (Fig. 3C), although the mRNA levels of CYP17A1 in the SOY group significantly increased compared to ND group. And there was no significant difference in protein levels about 3β-HSD among the two groups. In summary, increasing intake of soybean oil promoted the expression of SF-1 to enhance the transcription of StAR and CYP11A1, promoted the expression of them further, and then increased the synthesis of testosterone.

### Increasing intake of soybean oil up‐regulated the expression of LHCGR in the mice

**Fig. 4:**
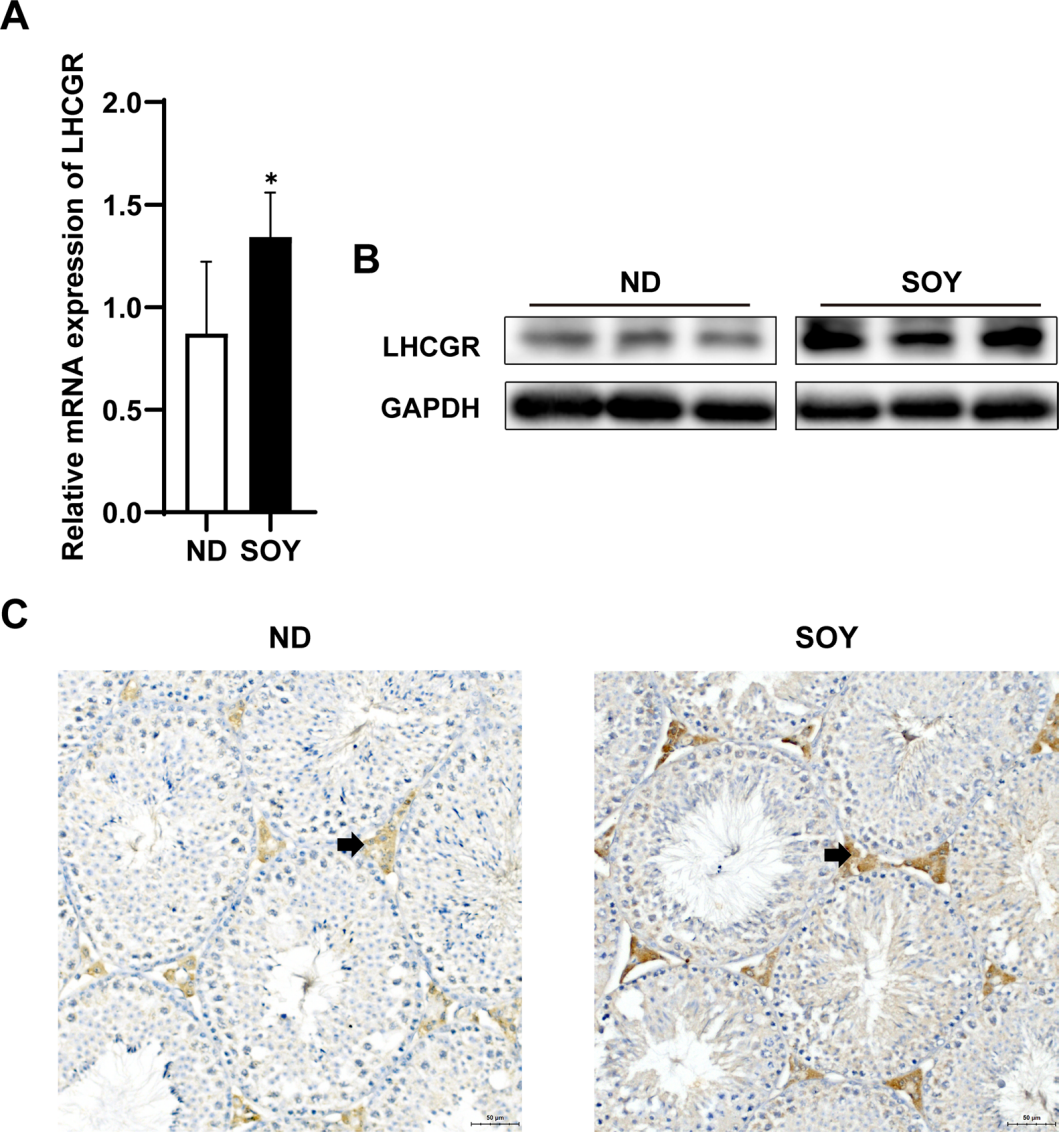
Increasing intake of soybean oil up‐regulated the expression of LHCGR in mice. **A** RT‐PCR analysis of the expression of LHCGR mRNA in testes of mice in the ND or SOY group (n = 6 for each group). **B** Immunoblot and quantitative analysis of LHCGR protein in testes of mice in the ND or SOY group (n = 3 for each group). **C** Testis was immunostained for LHCGR which is expressed in Leydig cells (n = 3 for each group). Data are expressed as mean ± SD (*P < 0.05)

We observed that the serum LH levels in the SOY group increased. Increasing intake of SOY may promote testosterone synthesis by high LH levels. LH need to combine with the LHCGR on the Leydig cell to promote testosterone production. Therefore, we used RT-PCR, immunoblotting analysis and immunohistochemistry to observe the effects of soybean oil on LHCGR. As Fig. 4A shown, compared with the ND group, the LHCGR mRNA levels of SOY group had a significant increase (*P* < 0.05). Immunoblotting analysis showed that the protein expression of LHCGR in SOY group increased (Fig. 4B). As expected, immunohistochemistry showed that the expression abundance of LHCGR in testicular Leydig cells in the SOY group was increased in comparison with the quantity of those in the ND group (Fig. 4C). In summary, increasing intake of soybean oil not only increases the level of LH in serum, but also up-regulates the expression of LHCGR in Leydig cells. The changes in hormones and their receptors both affect the production of testosterone.

## Discussion

It is well known that testosterone plays an important role in male development, maintenance of secondary sexual characteristics, sperm production, energy metabolism and many other functions [20, 21]. Many factors, such as unhealthy diet, chronic illnesses etc., all affect the level of testosterone [22].And the decline in serum testosterone has been suggested to be associated with infertility, diminished energy, muscle strength and physical function [23]. Therefore, it is meaningful to explore the causes of testosterone decline and the methods to improve testosterone levels. In recent years, with the changes in the dietary composition, the intake of soybean oil increased. But the effect of increasing intake of soybean oil on testosterone is still not clear. In this study, we demonstrated that increasing intake of soybean oil could improve the serum LH levels and up-regulate the LHCGR. Subsequently, accompanied by an increase in steroidogenic enzyme expression, the production of testosterone was promoted.

In order to study the effect of increasing intake of soybean oil on testosterone production, we added 20% soybean salad oil to the diet. There was also no statistical difference in glucose and serum lipids in the SOY group. It is possible that increasing intake of soybean oil did not form an obesity model with hyperlipidemia and hyperglycemia. In order to observe the effect of intake of soybean oil on the fatty acid composition, we analyzed the serum by gas chromatography. The main component of soybean oil is LA, and consistent with that, the serum LA of the mice in SOY group increased significantly. ALA is abundant in soybean oil [24]. Compared with ND group, the serum ALA of SOY group was significantly increased. The major components of soybean oil are PUFAs. Compared with the ND group, increasing intake of soybean oil reduced the levels of serum saturated fatty acids, especially PA. In agreement with some researches, soybean oil could increase serum LA and ALA levels and decrease saturated fatty acid levels [25–27]. Increasing intake of soybean oil changed the composition of serum fatty acids and that might influence the process of testosterone production.

Dietary LA plays an important role in regulating the balance between inflammation and anti-inflammation and steroid metabolism [28, 29]. A cross-sectional study indicated that a positive association was observed between the intake of PUFAs, particularly of ω-6 PUFAs, and LH concentrations [30]. It also has been reported that the addition of LA to porcine pituitary cells increased the release of LH [31]. We speculate that soybean oil promotes LH secretion mainly through the increased serum LA levels. Xu A et al. suggested that LA activates the GPR120 / ERK pathway in Leydig cells to promote the production of testosterone directly [32]. In this study, we speculate that in addition to the direct effects of LA on the testis, the testosterone production increased may be the result of the indirect effects of elevated LH levels. We believe that it is at least part of the LH of high levels acts on up-regulated LHCGR that activate the process of testosterone synthesis.

Some findings indicated that feeding of ω-3 PUFAs enriched diet could increase testosterone secretion and improve semen quality [33–36]. ALA, a kind of ω-3 PUFAs, plays an important role in regulating animal reproduction. Qi X et al. suggested ALA could increase the testosterone secretion, which may be related to higher StAR and CYP11A1 mRNA expression and SF-1 expression [37]. Soybean oil may promote testosterone synthesis by increasing serum ALA levels.

PA, a kind of saturated fatty acids, could induce apoptosis in testicular Leydig cells and affect testosterone synthesis [38–40]. As this study observed, increasing intake of soybean oil decreased the serum PA levels, which could contribute to the rising of testosterone.

Through the regulating activity of the hypothalamic-pituitary–gonadal axis, the testes exert endocrine functions to produce testosterone [41]. LH acts on Leydig cells to promote the synthesis of testosterone, so we tested the serum LH levels. Compared with the ND group, serum LH levels in mice of the SOY group increased. The up-regulation of LHCGR in the SOY group was more conducive to the function of LH. LH activated testosterone production, and compared with the ND group, the mRNA levels of SF-1, StAR, CYP11A1, and CYP17A1, the protein expression of StAR and CYP11A1 in the SOY group were all significantly increased. The mRNA levels of CYP17A1 in the SOY group significantly increased compared to ND group, but no significant difference about CYP17A1 at the protein levels is discovered. Protein is the working molecules of life activities. In this study, we believe that CYP17A1 may not play a role in increasing testosterone levels. The mRNA levels and protein levels are not always positively correlated in life activities, and the translation of mRNA into protein involves multiple life-regulating processes. CYP17A1 protein levels may be regulated by other processes without change. Consistent with McVey MJ's results, the intake of soybean oil can increase testosterone levels [42]. This study suggested that the increased serum LA and ALA and the decreased serum PA levels affected the above process. The underlying mechanisms need to be further studied.

Testosterone levels affects men's health. Low testosterone levels are associated with an increased fat mass, reduced insulin sensitivity, impaired glucose tolerance, elevation of triglycerides, and cholesterol and low HDL-cholesterol [43–47]. All these factors are found in the metabolic syndrome and type 2 diabetes, contributing to cardiovascular risk [21, 48]. Discussing the effects of testosterone on cardiovascular (CV) health is an ongoing debate. The overall evidence suggests that normal physiologic levels of testosterone are beneficial to the male CV system, and that testosterone deficiency is associated with an unfavorable metabolic profile and increased cardiovascular disease(CVD) events, such as myocardial infarction and mortality [49]. Clinical trials demonstrate that testosterone replacement therapy improves the insulin resistance and glycaemic control, and also reduces body fat mass in particular truncal adiposity, cholesterol, and triglycerides [21]. However, high testosterone levels can also compromise survivorship by increasing risk of prostate cancer, production of oxygen radicals, reduced tissue and organ maintenance, negative energy balance from adipose tissue catabolism, and suppression of immune functions [50]. The relationship between testosterone levels and disease is complicated. In this study, increasing intake of soybean oil raised testosterone levels. This change may reduce the occurrence of clinical disease states of obesity, metabolic syndrome, type 2 diabetes, and CVD, but it also could be associated with an increased risk of other disease. The further studies are required.

In conclusion, we demonstrated that increasing intake soybean oil could raise LH levels, up-regulate LHCGR, improve the steroid synthesis function of Leydig cells, and finally lead to the elevated testosterone levels. Because of the complexity of various fatty acids existing in soybean oil, we speculated that this effect may be caused by a kind of fatty acid alone, and it is more likely to be a comprehensive effect. That proposes a new potential pathway for the treatment of testosterone deficiency. However, further work should be carried out to unveil the underlying molecular mechanisms of the LH increasing and LHCGR up-regulation. Studies about the subsequent effects of elevated testosterone levels are also needed.

## Conclusion

This study suggests that increasing intake of soybean oil increased the serum LA and ALA levels and decreased serum PA levels, which could activate the LH/LHCGR pathway, improve the function of steroid synthesis in Leydig cells and finally lead to the elevated testosterone levels.

## Supplementary Information

**Additional file 1**: **Supplemental Table 1**. Ingredients of normal diet.

**Additional file 2: Supplemental Table 2**. Analysis of the content of fatty acid in the soybeanoil.

**Additional file 3: Supplemental Table 3**. Analysis of the content of fatty acid in the diets.

## Data Availability

The datasets used and/or analysed during the current study are available from the corresponding author on reasonable request.

## Abbreviations

SOY: Soybean oil
ND: Normal diet
LA: Linoleic acid
ALA: α-Linolenic acid
PA: Palmitic acid
PUFAs: Polyunsaturated fatty acids
FA: Fatty acid
T: Testosterone
LH: Luteinizing hormone
LHCGR: Luteinizing hormone/chorionic gonadotropin receptor
StAR: Steroidogenic acute regulatory protein
CYP11A1: Cytochrome P450 family 11 subfamily A member I
3β-HSD: 3β-Hydroxysteroid dehydrogenase
CYP17A1: Cytochrome P450 family 17 subfamily A member 1
SF-1: Steroidogenic factor 1
TG: Triglyceride
TC: Total cholesterol
LDL-C: Low-density lipoprotein cholesterol
HDL-C: High-density lipoprotein cholesterol
Glu: Glucose
ELISA: Enzyme-linked immunosorbent assay
