# Supplementary material for: Effects of increasing intake of soybean oil on synthesis of testosterone in Leydig cells
Source: Nutr Metab (Lond). 2021 May 26;18:53. doi: 10.1186/s12986-021-00580-1 (PMC8157704; doi:10.1186/s12986-021-00580-1)
Supplement: Supplementary file 1 — Additional file 1: Supplemental Table 1. Ingredients of normal diet. [file 12986_2021_580_MOESM1_ESM.docx]

Supplemental Table 1. Ingredients of normal diet

| Moisture | ≤10 | % | Sodium | 3.10 | g/kg |
| --- | --- | --- | --- | --- | --- |
| Crude protein | ≥18 | % | Magnesium | 2.90 | g/kg |
| Crude fat | ≥4 | % | Potassium | 7.40 | g/kg |
| Crude fiber | ≤5 | % | Copper | 11.40 | mg/kg |
| Coarse ash | ≤8 | % | Iron | 113.70 | mg/kg |
| Calcium | 1.0-1.8 | % | Manganese | 80.00 | mg/kg |
| Phosphorus | 0.6-1.2 | % | Zinc | 31.60 | mg/kg |
| Vitamin A | 7800.00 | IU/kg | Selenium | 0.20 | mg/kg |
| Vitamin D | 1200.00 | IU/kg | Iodine | 0.70 | mg/kg |
| Vitamin E | 67.00 | mg/kg | Methionine and Cystine | 5.80 | g/kg |
| Vitamin K | 5.00 | mg/kg | Lysine | 8.90 | g/kg |
| Vitamin B1 | 10.00 | mg/kg | Tryptophan | 2.10 | g/kg |
| Vitamin B2 | 15.00 | mg/kg | Arginine | 9.90 | g/kg |
| Vitamin B6 | 10.00 | mg/kg | Leucine | 14.80 | g/kg |
| Vitamin B12 | 0.02 | mg/kg | Isoleucine | 7.40 | g/kg |
| Niacin | 55.00 | mg/kg | Threonine | 6.60 | g/kg |
| Pantothenic acid | 22.00 | mg/kg | Valine | 8.90 | g/kg |
| Biotin | 0.20 | mg/kg | Histidine | 4.90 | g/kg |
| Choline | 1250.00 | mg/kg | Phenylalanine and Tyrosine | 14.60 | g/kg |
| Folic acid | 6.60 | mg/kg |  |  |  |
