# Supplementary material for: Effects of increasing intake of soybean oil on synthesis of testosterone in Leydig cells
Source: Nutr Metab (Lond). 2021 May 26;18:53. doi: 10.1186/s12986-021-00580-1 (PMC8157704; doi:10.1186/s12986-021-00580-1)
Supplement: Supplementary file 2 — Additional file 2: Supplemental Table 2. Analysis of the content of fatty acid in the soybeanoil. [file 12986_2021_580_MOESM2_ESM.docx]

Supplemental Table 2. Analysis of the content of fatty acid in the soybean oil

| FA (μg/mL) | Soybean oil |
| --- | --- |
| Saturated FA |  |
| C14:0 (Myristic acid) | 139.65 |
| C16:0 (PA) | 2581.33 |
| C18:0 (Stearic acid) | 1988.65 |
| C20:0 (Arachidic acid) | 604.36 |
| C22:0 | 658.97 |
| Total saturated FA | 5972.96 |
| Monounsaturated FA | |
| C14:1 | ND |
| C16:1 (Palmitoleic Acid) | 150.62 |
| C18:1 (Oleic acid) | 3800.13 |
| C20:1 | 396.68 |
| C22:1 (Erucic acid) | 64.66 |
| C24:1 | 30.94 |
| Total monounsaturated FA | 4443.03 |
| ω-6 Polyunsaturated FA |  |
| C18:2 ω-6 (LA) | 5221.49 |
| C18:3 ω-6 (γ-Linolenic acid) | 658.58 |
| C20:3 ω-6 | 18.84 |
| C20:4 ω-6 (AA) | 13.62 |
| Total ω-6 polyunsaturated FA | 5912.53 |
| ω-3 Polyunsaturated FA |  |
| C18:3 ω-3 (ALA) | 2754.38 |
| C20:3 ω-3 | 16.41 |
| C20:5 ω-3 (EPA) | 51.11 |
| C22:6 ω-3 (DHA) | 11.19 |
| Total ω-3 polyunsaturated FA | 2833.09 |

**Supplemental Table.2.** Analysis of the content of fatty acid (FA) in the soybean oil. ND: Not detected; PA: Palmitic acid; LA: Linoleic acid; AA: Arachidonic acid; ALA: α-linolenic acid; EPA: Eicosapentaenoic acid; DHA: Docosahexaenoic acid.
