# Supplementary material for: Effects of increasing intake of soybean oil on synthesis of testosterone in Leydig cells
Source: Nutr Metab (Lond). 2021 May 26;18:53. doi: 10.1186/s12986-021-00580-1 (PMC8157704; doi:10.1186/s12986-021-00580-1)
Supplement: Supplementary file 3 — Additional file 3: Supplemental Table 3. Analysis of the content of fatty acid in the diets. [file 12986_2021_580_MOESM3_ESM.docx]

Supplemental Table 3. Analysis of the content of fatty acid in the diets

| FA (μg/g) | Group | | |
| --- | --- | --- | --- |
|  | ND |  | SOY |
| Saturated FA |  | | |
| C14:0 (Myristic acid) | 63.46 |  | 107.30 |
| C16:0 (PA) | 1691.48 |  | 2060.30 |
| C18:0 (Stearic acid) | 857.39 |  | 1353.28 |
| C20:0 (Arachidic acid) | 41.29 |  | 297.26 |
| C22:0 | 25.03 |  | 340.41 |
| Total saturated FA | 2678.65 |  | 4158.55 |
| Monounsaturated FA | | | |
| C14:1 | 6.46 |  | 3.82 |
| C16:1 (Palmitoleic Acid) | 126.39 |  | 83.83 |
| C18:1 (Oleic acid) | 1963.35 |  | 2848.05 |
| C20:1 | 47.21 |  | 217.90 |
| C22:1 (Erucic acid) | 20.01 |  | 41.34 |
| C24:1 | 20.38 |  | 19.08 |
| Total monounsaturated FA | 2183.81 |  | 3214.01 |
| ω-6 Polyunsaturated FA |  | | |
| C18:2 ω-6 (LA) | 4056.42 |  | 4382.38 |
| C18:3 ω-6 (γ-Linolenic acid) | 11.39 |  | 32.35 |
| C20:3 ω-6 | 7.07 |  | 6.15 |
| C20:4 ω-6 (AA) | 33.04 |  | 14.44 |
| Total ω-6 polyunsaturated FA | 4107.92 |  | 4435.31 |
| ω-3 Polyunsaturated FA |  | | |
| C18:3 ω-3 (ALA) | 389.72 |  | 1657.17 |
| C20:3 ω-3 | 2.84 |  | 7.89 |
| C20:5 ω-3 (EPA) | 58.83 |  | 35.13 |
| C22:6 ω-3 (DHA) | 67.03 |  | 19.31 |
| Total ω-3 polyunsaturated FA | 518.43 |  | 1719.49 |

**Supplemental Table.3.** Analysis of the content of fatty acid (FA) in the diets. PA: Palmitic acid; LA: Linoleic acid; AA: Arachidonic acid; ALA: α-linolenic acid; EPA: Eicosapentaenoic acid; DHA: Docosahexaenoic acid.
